# Zingiber Officinale Alters 3,4-methylenedioxymethamphetamine-Induced Neurotoxicity in Rat Brain

**Published:** 2012-12-12

**Authors:** Mehdi Mehdizadeh, Fataneh Dabaghian, Akram Nejhadi, Hassan Fallah-huseini, Samira Choopani, Nima Shekarriz, Nima Molavi, Arghavan Basirat, Farzaneh Mohammadzadeh Kazorgah, Alireza Samzadeh-Kermani, Sara Soleimani Asl

**Affiliations:** 1. Research Institute for Islamic and Complementary Medicine (RICM), Tehran University of Medical Sciences, Tehran, Iran; 2. Department of Anatomy, Faculty of Medicine, Tehran University of Medical Sciences, Tehran, Iran; 3. Department of Anatomy, Faculty of Medicine, Army University of Medical Sciences, Tehran, Iran; 4. Institute of Medicinal Plants, Jihad University (ACECR), Tehran Province, Tehran, Iran; 5. Department of Physiology and Pharmacology, Pasteur Institute, Tehran, Iran; 6. Faculty of Medicine, Tehran University of Medical Sciences, Tehran, Iran; 7. Department of Chemistry, Faculty of Sciences, Zabol University, Zabol, Iran; 8. Department of Anatomy, Faculty of Medicine, Hamadan University of Medical Sciences, Hamadan, Iran; 9. Research Center for Behavioral Disorders and Substance Abuse, Hamadan University of Medical Sciences, Hamadan, Iran

**Keywords:** Apoptosis, Ginger, Spatial Memory, MDMA, Hippocampus, Bcl-2 Family

## Abstract

**Objective:**

The spice Zingiber officinale or ginger possesses antioxidant activity and neuroprotective effects. The effects of this traditional herbal medicine on 3,4-methylenedioxymethamphetamine (MDMA) induced neurotoxicity have not yet been studied. The present study considers the effects of Zingiber officinale on MDMA-induced spatial memory impairment and apoptosis in the hippocampus of male rats.

**Materials and Methods:**

In this experimental study, 21 adult male Sprague Dawley rats (200-250 g) were classified into three groups (control, MDMA, and MDMA plus ginger). The groups were intraperitoneally administered 10 mg/kg MDMA, 10 mg/kg MDMA plus 100 mg/kg ginger extract, or 1 cc/kg normal saline as the control solution for one week (n=7 per group). Learning memory was assessed by Morris water maze (MWM) after the last administration. Finally, the brains were removed to study the cell number in the cornu ammonis (CA1) hippocampus by light microscope, Bcl-2 by immunoblotting, and Bax expression by reverse transcription polymerase chain reaction (RT-PCR). Data was analyzed using SPSS 16 software and a one-way ANOVA test.

**Results:**

Escape latency and traveled distances decreased significantly in the MDMA plus ginger group relative to the MDMA group (p<0.001). Cell number increased in the MDMA plus ginger group in comparison to the MDMA group. Down-regulation of Bcl-2 and up-regulation of Bax were observed in the MDMA plus ginger group in comparison to the MDMA group (p<0.05).

**Conclusion:**

Our findings suggest that ginger consumption may lead to an improvement of MDMA-induced neurotoxicity.

## Introduction

3,4-methylenedioxymethamphetamine (MDMA) is known to produce brain damage and spatial memory impairment (1, 2). Our recent studies have shown that MDMA causes spatial memory impairment (3) and multiple doses of MDMA can induce cell death through an apoptotic pathway that implicates up-regulation of Bax and down-regulation of Bcl-2 in the hippocampus of male rats (4). Oxidative stress responses involve MDMA-induced neurotoxicity that lead to the formation of hydroxyl radicals (5), lipid peroxidation (6), and an increase in the number of tunnel-positive cells in the hippocampus. MDMA induces cell death trough an apoptotic pathway by releasing cytochrome c and activation of the caspase cascade (7). MDMA treatment also results in a decrease in intracellular glutathione (GSH) and neural death (8).

There is evidence that dietary enrichment with nutritional antioxidants could improve brain damage and cognitive function (9, 10). Zinger officinale roscoe or ginger, a member of the Zingiberaceae family, is widely used as a spice. It is used in traditional Asian medicine for the treatment of stomach aches (11), nausea, diarrhea, and joint and muscle pain (12).

Recently, several research groups have demonstrated that ginger has antioxidant activity (13, 14) and a neuroprotective effect (15). Another study suggests that ginger can reduce cell death and restore motor function in a rat spinal cord injury (16). We hypothesize that if ginger could scavenge free radicals, an important factor in producing brain damage induced by MDMA, it might also be able to improve spatial memory impairment related to the MDMA group via reduction of oxidative stress. Secondly, if ginger has any apoptotic effect, then MDMA plus ginger-treated rats should exhibit diminished apoptotic factors in the hippocampus in comparison to MDMA-treated rats. This study assesses the effectiveness of ginger on MDMA-induced neurotoxicity in the hippocampus of adult rats.

## Materials and Methods

### 3, 4-methylenedioxymethamphetamine and ginger preparation

MDMA was obtained from the Presidency Drug Control Headquarters. Zingiber officinale rhizomes (herbarium code no. 1483) were collected from a field at the Iranian Institute of Medicinal Plants. Approximately 500 g of the dried rhizome powder from Zingiber officinale were extracted with 3 liters of 70% aqueous ethanol using the percolation method at room temperature. The extracts were filtered through Whatman filter paper and evaporated to dryness under reduced pressure at a maximum of 40℃ using a rotary evaporator. Zingiber officinale yielded 33.28% dried extract.

### Animals

We obtained 21 adult male Sprague Dawley rats that weighed 200-250 g from the Iranian Razi Institute. The rats were allowed to acclimatize to the colony room for one week prior to the MDMA administration. As MDMA causes hyperthermia, any animal with an elevated body temperature was excluded. The rats were maintained in the colony room at a temperature of 21 ± 1℃ (50 ± 10% humidity) on a 12 hour light/12 hour dark cycle with access to water and food ad libitum. All experimental procedures were performed in accordance with the Guidelines of the Ethical Committee of Tehran University of Medical Sciences.

The 21 rats were assigned to the following groups: i. sham group (n=7) received normal saline (1 cc/kg) intraperitoneal (IP) injections daily for one week; ii. MDMA group (n=7) received 10 mg/kg MDMA (IP) daily for one week; and iii. treatment group (n=7) received IP injections of ginger (100 mg/kg) at 9:00 plus MDMA (10 mg/kg) at 13:00, daily for one week.

Learning memory was assessed by the Morris water maze (MWM) the day following the last administration.

### Morris water maze performance

MWM was used for assessment of spatial memory and included a circular pool (136 cm in diameter, 60 cm in height) that was painted black and filled to a depth of 25 cm with water at a temperature of 22 ± 1℃. The pool was divided into four quarters with four starting locations: north (N), south (S), east (E), and west (W) located at equal distances on the rim.

An invisible platform (10 cm in diameter) made of Plexiglass was located 1 cm below the water in the center of the northern quadrant. The animals were trained for three days at approximately the same time (10-12 am) each day. Each training day included two blocks with four trials. The time limit on each animal was 90 seconds and the intertrial was 30 seconds that was spent on the platform. The rats rested for 5 minutes between two consecutive blocks.

A video camera mounted directly above the water maze pool was linked to a computer and recorded the time to reach the hidden platform (escape latency), the length of swim path (traveled distances), and percentage of time spent in the target quarter for each rat. The day after the last learning trial, each rat was given a single 60 second probe trial and visible test. The probe trials were performed without a platform and the visible tests were performed with a platform that was covered with aluminum foil.

### Histological procedure

For light microscopic study, sections were prepared using our previously described method (17).Rats were perfused with 4% paraformaldehyde in 0.1M phosphate buffer (pH =7.3) and the hippocampi were serially sectioned into 10 µm coronal sections. After deparaffinization and rehydration, the sections were stained in 0.1% cresyl violet for 3 minutes. Finally, the sections were photographed with a digital camera (Olympus, DP 11, Japan) attached to a microscope (Olympus Provis, Ax70, Japan). For each animal, average neuronal counts were obtained by counting five serial coronal sections at 120 µm intervals using ×400 magnification. Only complete neuronal cells that had clearly defined cell bodies and nuclei were counted.

### Western blot experiment

Animals were killed by cervical dislocation and the brains were rapidly removed. The hippocampi were dissected out on ice and then frozen in liquid nitrogen and maintained at -80℃ until used. The frozen hippocampi were homogenized with ice-cold lysis buffer (Ripa buffer with a protease inhibitor cocktail at a 1:10 ratio) for 1 hour and centrifuged (Eppendorf, Hamburg, Germany) at 12000 g for 20 minutes at 4℃. The supernatant was removed and conserved. After determining the protein concentration by Bio-Rad assay (Bio-Rad, San Francisco, CA, USA), aliquots of 100 µg of protein from each sample were denatured with the sample buffer (6.205 mM tris-HCl, 10% glycerol, 2% SDS, 0.01% bromophenol blue, and 50 mM 2-ME) at 95℃ for 5 minutes and separated on 10% sodium dodecyl sulfate polyacrylamide gel electrophoresis (90 minutes, 120 voltage). The proteins were then transferred to a Hybond-PTM membrane (Amersham Pharmacia Biotech, Piscataway, New Jersey, USA).

Membranes were blocked with 5% nonfat milk dissolved in TTBS buffer (tris 50mM, NaCl 1.5% and 0.05% of tween 20, pH=7.5) for 1 hour. Membranes were stained with anti-Bcl-2 and anti-Bax monoclonal antibody (1:1000 Sigma Aldrich, Saint Louis, MO, USA) for 2 hours followed by secondary antibody alkaline phosphatase-conjugated anti-mouse antibodies (1:10000, Sigma Aldrich, Saint Louis, MO, USA) for 1 hour. Bands were detected by chromogenic substrate 5-bromo-4-chloro-3-indolyl phosphate in the presence of nitroblue tetrazolium. β-actin antibody (1:1000, Sigma Aldrich, Saint Louis, MO, USA) was used to detect endogenous standard for normalization. The bands from various groups which corresponded to the appropriate molecular weight for each subunit were analyzed and values were compared using densitometric measurements by an image analysis system (UVIdoc, Houston, Texas, USA).

### Reverse transcriptional-PCR experiment

Total mRNA was extracted from frozen hippocampi by phenol-chloroform. Tissue samples were homogenized in 1000 µl RNATM (Cinnagen, Tehran, Iran), then 200 µl of ice-cold chloroform was added. The homogenates were centrifuged (Eppendrof, Hamburg, Germany) at 12000 g for 20 minutes at a temperature of 4℃. The RNA of the water-soluble supernatant was precipitated with isopropanol and washed with 75% ethanol The air-dried RNA pellet was dissolved in RNase-free water. cDNA first-strand synthesis was performed by a cDNA Synthesis Kit (Quiagen, Hilden, Germany) following the protocol outlined by the company. First strand cDNA (2.5 ml) was used as template for subsequent PCR with a PCR Master Kit (Cinnagen, Tehran, Iran) and primers (Cinnagen, Tehran-Iran) as follows:

β-actin:
  forward:5'-TGGAGAAGAGCTATGAGCTGCCTG -3'
  reverse:5'-GTGCCACCAGACAGCACTGTGTTG -3'
Bax:
  forward5'-CCAAGAAGCTGAGCGAGTGTCTC -3'
  reverse:5'-AGTTGCCATCAGCAAACATGTCA -3'
Bcl-2:
  forward: 5'- CGCCCGCTGTGCACCGAGA -3'
  reverse: 5'- CACAATCCTCCCCCAGTTCACC -3'

For PCR, 1 µl of cDNA was placed into a 24 µl reaction volume that contained 12.5 µl Master Mix, 1 µl of each primer, and 9.5 µl of sterile deionized water. The PCR reactions included an initial denaturation at 95℃ for 3 minutes, followed by 31 cycles at 95℃ for 20 seconds, 65℃ for 30 seconds, and 72℃ for 30 seconds for Bax; and 35 cycles at 95℃ for 30 seconds, 60℃ for 1 minute, and 72℃ for 60 minutes for Bcl-2. The reactions were terminated at 72℃ for 7 minutes as the elongation period. The same annealing temperature was used for β-actin. PCR products were separated by electrophoresis in 1.5% agarose gel at 100 V.

Semi-quantitative analysis was assessed by a digital imaging system (UVIdoc, Houston, Texas, USA).

### Statistical analysis

The data were presented as the mean ± SD and results were analyzed by SPSS 16 software and one-way ANOVA. Post-hoc comparisons were performed using Tukey’s test. P≤ 0.05 was considered statistically significant.

## Results

### Protective effect of ginger on learning memory in Morris water maze

**Fig 1:**
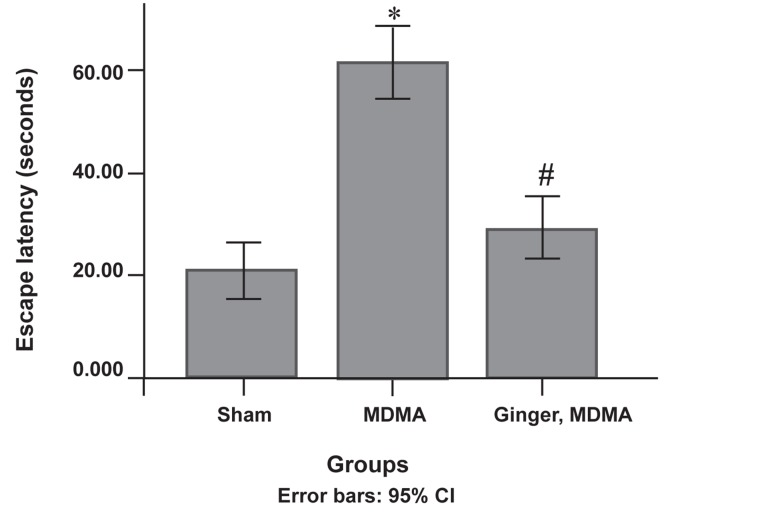
The protective effect of ginger in MDMA-induced escape latency impairment during the training days. After last administration, escape latency was analyzed by using the Morris water maze (MWM). Data are presented as mean ± SD (n=7). Differences between the groups that were significant: *; p<0.001, MDMA vs. sham group. #; p<0.001, ginger plus MDMA group vs. MDMA group.

**Fig 2:**
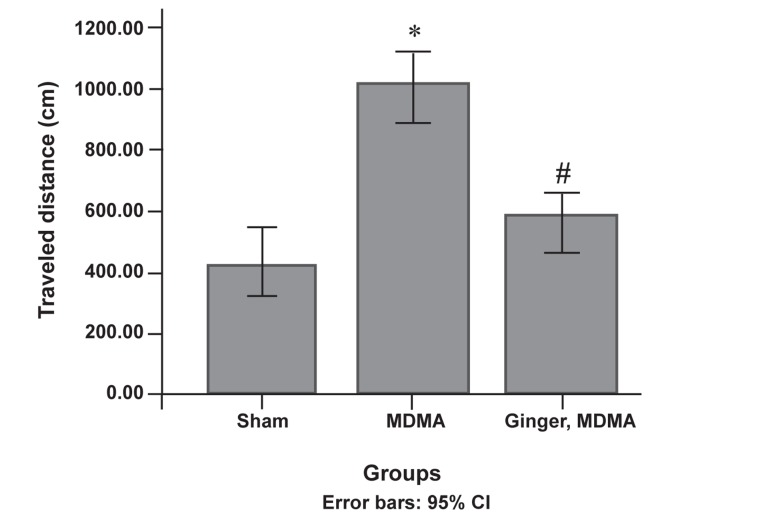
Protective effect of ginger on MDMA-induced traveled distance increase during the training days. After the last administration, distance traveled was assessed using MWM. Data are presented as mean ± SD (n=7). Differences between the groups that were significant: *; p<0.001, MDMA vs. sham group. #; p<0.001, ginger plus MDMA group vs. MDMA group.

**Fig 3:**
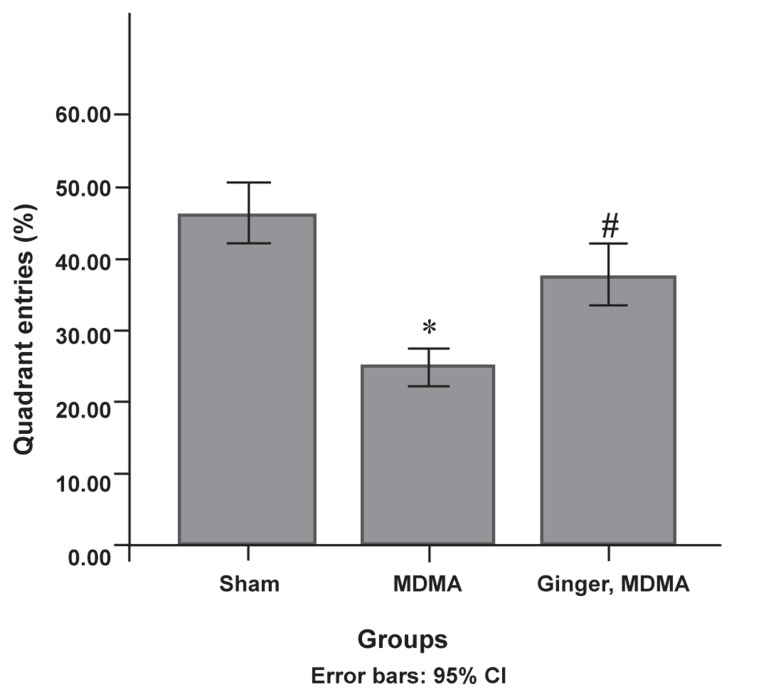
Protective effect of ginger on MDMA-induced quarter entrance decrease on the third day of training. Following the final administration, quarter entrance was assessed using the Morris water maze (MWM). Data are presented as mean ± SD (n=7). Differences between the groups that were significant: *; p<0.001, MDMA vs. sham group. #; p<0.001, ginger plus MDMA group vs. MDMA group.

There was a significant decrease in the escape latency mean in the treatment group compared to the MDMA group in the MWM during the training days (p<0.001, Fig 1). The treatment group showed a significant decrease in traveled distance to the escape platform compared to the MDMA group (p<0.0001, Fig 2). According to the results, the treatment group spent significantly more total time in the target quarter where the platform was located during the training days compared to the MDMA group (p<0.0001, Fig 3). Administration of ginger prior to MDMA caused a significant increase in escape latency and traveled distance, and a decrease in the presence of total time in target quarter when compared with the sham group (p<0.05 for all groups).

### Protective effect of ginger on neuronal density in CA1 hippocampus

**Fig 4:**
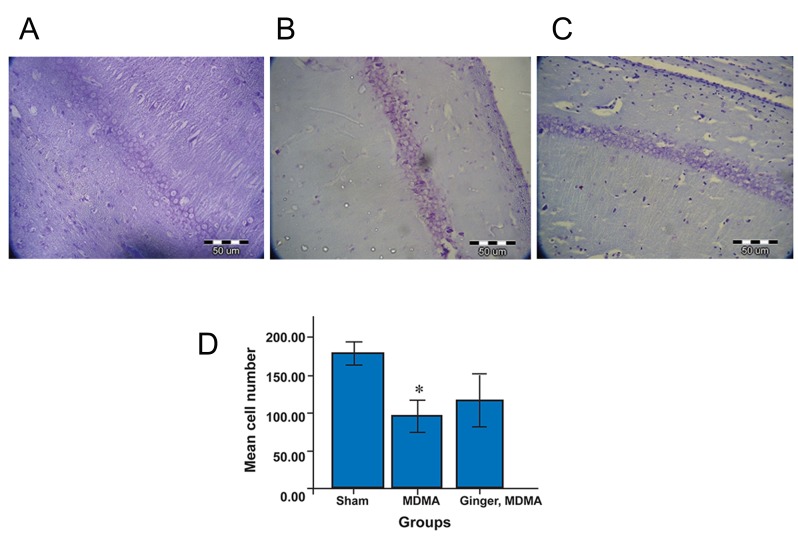
Nissl staining in sham (A), MDMA (B), and MDMA plus ginger groups (C). After MWM assessment, rat brains were removed and fixed. Hippocami were serially sectioned into 10 µm coronal sections and stained in 1% crystal violet. Scale bar=50 µm, magnification: ×40. (D) Protective effect of ginger on MDMA-induced cell death in CA1 hippocampus. Data are presented as mean ± SD: *; p<0.001, MDMA vs. sham group.

Our results showed that MDMA significantly decreased neuronal density in the CA1 hippocampus compared to the sham group (p<0.001, Fig 4). Although neuronal density increased in the treatment group (mean=118.00 ± 28.08) compared to the MDMA group (mean=96.20 ± 18.45), this increase was not significant (Fig 4).

### Protective effect of ginger on Bax and Bcl-2 protein expression

**Fig 5:**
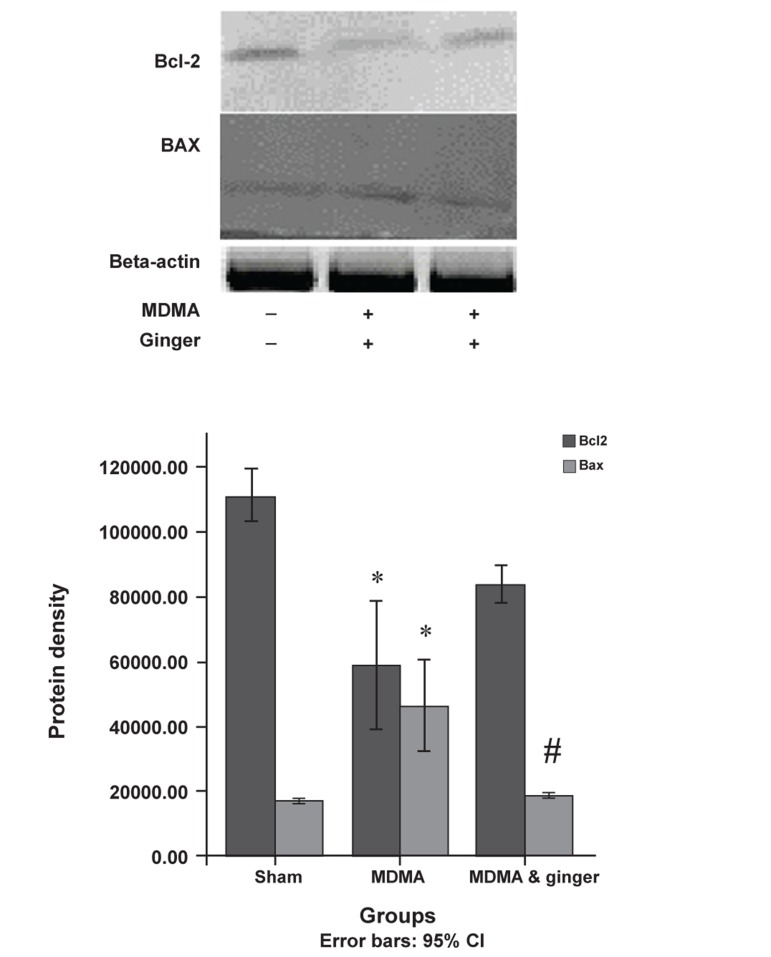
Western blot analysis of BAX and Bcl-2 protein expression in sham and treatment groups. The frozen hippocampi were lysed and transferred to nitrocellulose paper, then incubated with anti-Bcl-2, anti-Bax, and secondary anti-mouse antibodies. Bands were detected by chromogenic substrate. Data analyzed by one-way analysis of variance (ANOVA) followed by Tukey’s test for multiple comparisons. *; p<0.05, MDMA vs. sham group. #; p<0.05, ginger plus MDMA group vs. MDMA group.

MDMA caused up-regulation of Bax and down-regulation of Bcl-2 protein expression (p<0.05). Ginger significantly decreased Bax expression compared to the MDMA group (p<0.05, Fig 5). Ginger administration led to more expression of the Bcl-2 protein (mean=85.47 ± 55.18) compared to the MDMA group (mean=60.73 ± 18.96), however this was not significant (Fig 5).

**Fig 6:**
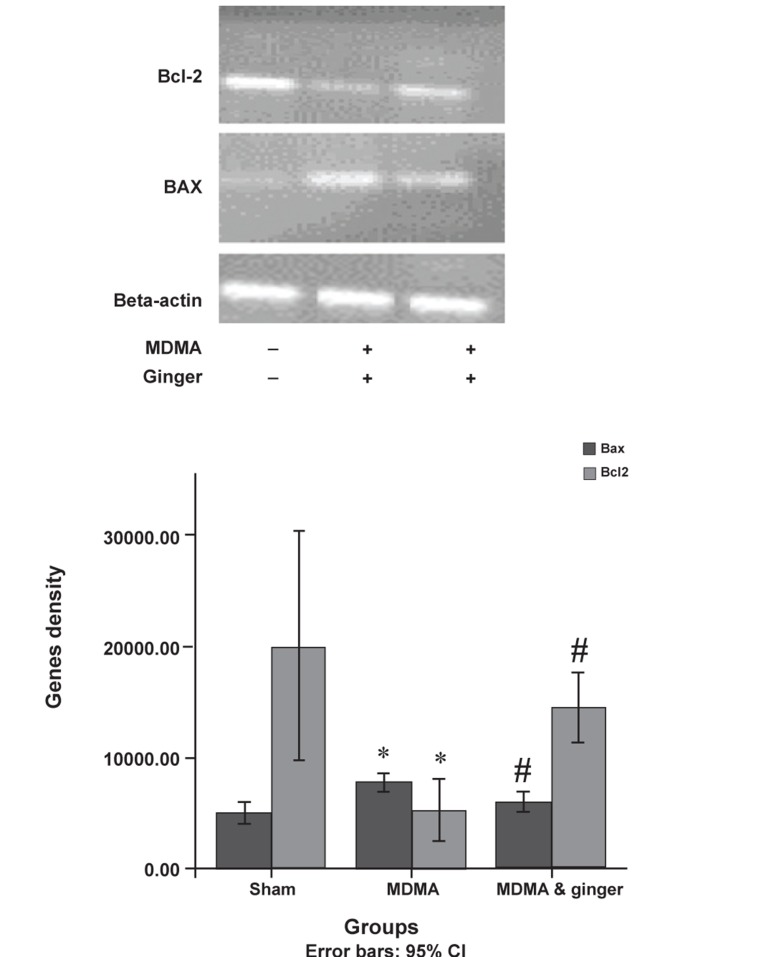
Bax and Bcl-2 gene expression in sham and treatment groups. Total RNA was extracted by phenol-chloroform. After cDNA synthesis, PCR was performed using the respective primers. Data were analyzed with one-way analysis of variance (ANOVA) followed by Tukey’s test for multiple comparisons. *; p<0.001, MDMA vs. sham group #; p<0.001, ginger plus MDMA group vs. MDMA group.

## Discussion

Our results demonstrated that Zingiber officinale could protect brain damage in MDMD treated rats. It also reduced learning memory deficits that were induced by MDMA.

MDMA has been reported to induce a number of important cellular changes, including an increase in free radicals that lead to brain damage and learning impairment (6, 18). Other studies have shown that Zingiber officinale rhizome extract improved cognitive function and neuronal density in the hippocampus of focal cerebral ischemic rats (19) and memory impairment in amyloid-beta protein-induced amnesia in mice (20), both consistent with our study results. The cognitive enhancing effect of Zingiber officinale occurs not only due to its ability to increase neuronal density in the hippocampus, but also due to other mechanisms such as induction of vasodilation (21). Therefore, it might be possible that Zingiber officinale enhances cerebral blood flow which would result in improvements of spatial memory. The hippocampus is an important brain structure associated with learning, memory, and cognition (22). Many neurotoxic factors (such as ischemia and MDMA) can induce neuronal damage in the hippocampus (4, 23, 24). MDMA treatment leads to rapid intracellular Ca^2+^ influx, mitochondrial membrane depolarization, ROS production, and caspase-9 activation (25). The injection of methamphetamine as another amphetamine derivative triggers activation of the programmed cell death pathway via up-regulation of Bax and down regulation of Bcl-2 (26).

Our results showed up-regulation and down-regulation of Bax and Bcl-2 genes (and subsequently their proteins) in the ginger plus MDMA treated group compared to the MDMA group. This was consistent another study that demonstrated 6-shagoal purified from Zingiber officinale lead to a prominent decrease in the PARP apoptosis protein and an increase in the expression of Bcl-2 anti-apoptotic protein in a spinal cord injury model (16). It has been reported that Zingiber officiale can decrease oxidative stress by increasing the activity of SOD, CAT, and GSH in the cerebral cortex and hippocampus, resulting in a decrease of the lipid peroxidation level in all areas mentioned earlier (19). Hanish Singh et al. (20) have shown that Zingiberaceae decreases the neurotransmitter metabolic enzyme AChE and increases the activity of Na^+^/K^+^-ATPase in amyloid-beta protein-induced neurotoxicity . It was shown that the neuroprotective effects of Zingiber officinale extract may be mediated through free radical scavenging activity, inhibition of cholinesterase, and pro-inflammatory cytokines.

## Conclusion

Our findings have shown that the administration of Zingiber officiale in MDMA-treated rats attenuated apoptotic cell death and improved learning memory. Thus, Zingiber officiale may be used as a therapeutic agent in the prevention of complications among MDMA users, however because of the hyperthermia effect of MDMA and also increased metabolism following the consumption of Zingiber officinale, additional assays need to be performed.
